# Genome Sequence of *Listeria monocytogenes* Strain F6540 (Sequence Type 360) Collected from Food Samples in Ontario, Canada

**DOI:** 10.1128/genomeA.01507-15

**Published:** 2016-01-14

**Authors:** Saravanamuttu Gnaneshan, Ya-Chih Hsueh, Lindsay Liang, Sarah Teatero, Nahuel Fittipaldi, Gustavo V. Mallo

**Affiliations:** a Public Health Ontario, Toronto, Canada; b Department of Laboratory Medicine and Pathobiology, University of Toronto, Toronto, Canada

## Abstract

Comparative genomic analysis between pathogenic and nonpathogenic *Listeria monocytogenes* strains provides a good model for studying the virulence of this organism. Here, we report the genome sequence of the nonpathogenic *L. monocytogenes* strain F6540 (sequence type 360) identified specifically in food samples in Ontario, Canada, in 2010.

## GENOME ANNOUNCEMENT

*Listeria monocytogenes* is naturally found in soil as a saprophyte, but it can also infect humans. It has been estimated that almost all human listeriosis cases are caused by the consumption of contaminated food products (1). Many studies have addressed the ability of *L. monocytogenes* to infect host organisms. However, to this date, no significant differences have been observed between isolates (2–4). The genomes of several clinical isolates of *L. monocytogenes* have been sequenced and are publicly available. Increasing *L. monocytogenes* genome data availability for strains isolated specifically from food is key to permitting comparative genomics and enhancing studies on the virulence of this pathogen. Here, we describe the draft genome sequence of *L. monocytogenes* strain F6540. This strain belongs to the multilocus sequence type (MLST) 360 (5) and was recovered from food samples in 2010 in Ontario, Canada. Characterization of an extensive collection (*n* = 350) of clinical samples recovered in Ontario from 2004 to 2011 using MLST could not identify ST360 among clinical *L. monocytogenes* cases.

Short-read sequences were generated with the Illumina HiSeq 2500 instrument (Illumina, San Diego, CA), according to the protocols from the manufacturer. Read quality was assessed with FastQC version 0.1 (http://www.bioinformatics.babraham.ac.uk/projects/fastqc/). Trimmomatic 0.30 (6) was used to preprocess the sample reads. Briefly, sequencing adapters, overrepresented sequences, and reads <80 bp were removed. The reads were trimmed to remove low-quality regions. A hybrid technique combining alignment and *de novo* assembly was used to assemble the *Listeria* genome. An initial alignment was performed using BWA 0.7.5a (7). The reads were aligned against all the available *Listeria* reference genomes from NCBI. *L. monocytogenes* SLCC7179 had the highest alignment score and was chosen as the reference genome. Next, we performed *de novo* assembly using Velvet 1.2.09 (8), with optimized parameters derived from VelvetOptimiser 2.2.5 (http://bioinformatics.net.au/software.velvetoptimiser.shtml). progressiveMauve 2.3.1 (9) was used to order and rearrange the contigs using *L. monocytogenes* SLCC7179 as the reference genome. A selection of the above-mentioned steps, in combination with manual curations, was repeated using sample reads preprocessed with a lower-quality threshold to reduce the number of contigs and gaps. The resulting assembly had a total length of 2,881,130 bp organized in 11 scaffolds, with 33.2-fold average sequencing coverage and 37.9% G+C content. Gene prediction and annotations were performed using the annotation tools from the Victorian Bioinformatics Consortium—Prokka 1.9 (10). A total of 2,846 coding sequences (CDSs), 49 tRNAs, and 3 rRNAs were identified. Analysis of virulence genes (a*ctA*, *hly*, *hpt*, *inlA*, *inlB*, *pclA*, *pclB*, *mpl*, and *pfrA*), did not show any early stop codons compared with the *L. monocytogenes* EGD-e reference strain (NCBI reference sequence: NC_003210), which might explain the loss of virulence of the F6540 strain. *In silico* analysis of the MLST target genes (*abcZ, bglA*, *cat*, *dapE*, *dat*, *ldh*, and *lhkA*) confirmed that this strain is related to lineage II, clonal complex 14 (CC14), sequence type 360 (ST360). Comparative genome analysis between *L. monocytogenes* pathogenic and nonpathogenic strains, like F6540, might help us better understand the mechanisms of this bacterium to infect humans.

### Nucleotide sequence accession numbers.

This whole-genome shotgun project was deposited at DDBJ/EMBL/GenBank under the accession number LMTM00000000. The version described in this paper is the first version, LMTM01000000.
